# Five-Year Long-Term Followup of a Primary Lymph node Gastrinoma: Is a Pancreaticoduodenectomy Justified?

**DOI:** 10.1155/2009/762791

**Published:** 2009-08-26

**Authors:** Bernd Jaenigen, Gian Kayser, Berthold Steinke, Oliver Thomusch

**Affiliations:** ^1^Department of General and Visceral Surgery, Albert-Ludwigs University of Freiburg, 79106 Freiburg, Germany; ^2^Institute of Pathology, Albert-Ludwigs University of Freiburg, 79106 Freiburg, Germany; ^3^Department of Internal Medicine, County Hospital Rottweil, 78628 Rottweil, Germany

## Abstract

*Background*. Gastrinoma-positive lymph nodes and failed localization of the primary tumor during surgical exploration are described. Specialists suppose that these lymph nodes are metastases rather than a primary gastrinoma. *Methods*. Case report with a five-year long-term followup. A 60-year-old patient with an confirmed gastrinoma was treated in our department. All preoperative evaluations including somatostatin-receptor-scintigraphy and F-Dopa PET failed to localize the gastrinoma. Explorative laparotomy revealed a gastrinoma in two peripancreatic lymph nodes. Despite extensive intraoperative exploration, no primary gastrinoma could be detected in typical localization. *Results*. Over a period of 5 years, the patient's gastrin level stayed in the normal range and the patient seems to be completely cured. *Conclusion*. A prophylactic partial pancreatoduodenectomy is not indicated to avoid recurrence, since complete biochemical cure by local resection of the lymph node gastrinoma is possible.

## 1. Introduction

 In 1955, Zollinger and Ellison described a new syndrome combining recurrent gastric and duodenal ulcers, excessive gastric acid secretion, and a noninsulin-secreting pancreatic tumor [1]. Today's comprehension of the Zollinger-Ellison-Syndrome (ZES) is more differentiated. The ZES has an incidence of 1 per million inhabitants per year [2]. The gender proportion shows a slight advantage in favour of the male. More than 90% of patients with ZES typically develop multiple gastric or duodenal ulcers, preferably located in the bulbus duodeni or the stomach. Abdominal pain (75–90%) and diarrhea (40–73%) are the most common symptoms [3]. Pyrosis (40–60%), nausea (28%), vomiting (26%), and weight loss (17%) are further typical clinical symptoms [4]. Only 11% of the patients suffered from a single symptom, and 55% of the patients complain about the combination of abdominal pain and diarrhea [3]. The ZES can be divided into two different entities. About 62–80% of the gastrinomas appear sporadically. The other 20–38% are associated with the hereditary (autosomal-dominant) MEN-I syndrome [3, 5–7] with additional endocrine tumours of the parathyroid gland (94%), the pituitary gland (60%), and the adrenal gland (45%) [8]. Tumour manifestations are localized mainly in the so-called gastrinoma triangle. There are three main differences between the two gastrinoma entities:

- localization of primary tumour [9],
- prognosis [9],
- age of the patients [3, 9].

Sporadic gastrinomas are equally localized in the duodenum and the pancreas, and are characterized by a solitary lesion [10]. The peak incidence of the sporadic gastrinoma is described between 40 and 50 years of age in contrast to the MEN-I-associated gastrinoma, which appears more likely in younger adults (between 30 and 40 years of age) [9]. The MEN-I-associated gastrinomas are mainly located in the duodenum [11]. Very often, patients suffer from multiple tumours. Overall, the long-term survival rate is better in MEN-I-associated gastrinomas [9]. Despite 10-year survival rates of 86% in medically treated patients, complete resection is associated with a further survival benefit (10-year survival rate 96%) [12, 13]. After biochemical proof (at least triple elevated serum gastrin and a positive secretin provocation test), exact preoperative localization of the tumour and the exclusion of distant metastases are strongly recommended before surgery. Several different diagnostic techniques are available. Specialists recommend the endosonography with a sensitivity of 40–100% as the method of choice for preoperative localization of the primary tumour in sporadic gastrinomas because of lower sensitivity of imaging techniques like the conventional abdominal ultrasonography (24–44%), computertomography (27–56%), and MRI (25–46%) [14–18]. A Somatostatin-receptor scintiscan with a sensitivity of 27–92% is essential to preoperatively exclude positive lymph node involvement, liver or distant metastases [15, 16, 18, 19]. The selective arterial Secretin injection test (SASI) with a sensitivity of 86–100% is suggested for patients with MEN-I-associated gastrinomas or recurrent disease [11, 15, 17], but this method can only regionalize the lesion. A promising new method is the F-Dopa PET. The F-Dopa PET seems to be superior to the other investigating techniques, but it is not yet well established [5, 20]. In comparison with the preoperative localisation, intraoperative localisation by palpation and intraoperative ultrasonography (IOUS) is successful in 96% [16].

A review of the literature was performed on the occasion of this case report with two gastrinoma-positive lymph nodes without any detected duodenal or pancreatic lesion. This overview discusses the existence of primary lymph node gastrinomas and adequate therapy regimens.

## 2. Case Report

In October 2003, a 60-year-old patient attended a department of internal medicine. The patient complained about pain in the upper abdominal region. A gastroscopy showed an extended inflammation of the duodenum and in the esophagus with multiple ulcers. During the hospital stay, a Forrest Ia bleeding took place in the duodenal region. The bleeding could be arrested endoscopically by clipping. A Zollinger-Ellison-Syndrome was suspected because of the extended duodenal and esophagal inflammation with multiple ulcers. The diagnosis was proven by measuring elevated values (1528 ng/L) of Gastrin (reference range lower 115 ng/L). The duodenal ulcers and the esophageal inflammation healed slowly under therapy with omeprazole. In order to exclude a MEN-I syndrome, 5-hydroxyindole acetic acid, parathyroid hormone, prolactine, and ACTH were determined. All parameters showed normal values.

**Figure 1:**
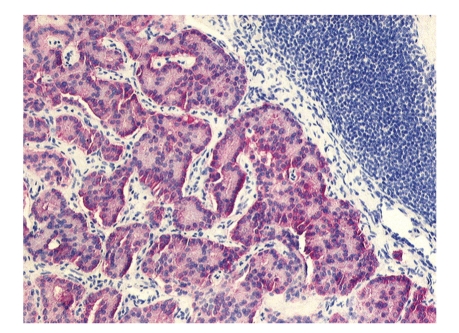
Trabecular epithelial cell proliferation with positive expression of synatophysin (10x).

An abdominal computer tomography was performed to locate the gastrinoma, but no suspected lesion could be detected. Additionally, a Somatostatin receptor scintiscan could also not determine the focus. The patient was presented in our department to plan further treatment. After a Dopa PET failed to localize the gastrinoma, an explorative laparotomy was performed in April 2004. First, the duodenum and the pancreas head were exposed by carrying out a Kocher's manoeuvre. Then, the pancreas corpus and tail were completely mobilized. Palpation and intraoperative ultrasonography showed a normal pancreas without any suspicious pancreatic tumour. An enlarged lymph node (1 × 0.5 cm) was removed from the retropancreatic region. Instantaneous section found glandular structures matching a gastrinoma. Subsequently. a complete lymphadenectomy of the retropancreatic area and the hepatoduodenal ligament was performed. A second enlarged lymph node was extirpated behind the common bile duct. Finally, a duodenotomy of pars II was performed. Despite careful examination, no duodenal lesion could be found and the surgery was completed. The histology of the specimen showed two lymph nodes infiltrated by a gastrinoma. An immunohistochemical preparation of the specimen showed a positive expression of gastrin, cytokeratin, chromogranin, and synaptophysin (see Figure 1).

The patient recovered well from surgery and was discharged on day 12. In May 2004, the patient underwent a followup examination in our endocrinology department. The serum gastrin was 120 ng/L. The patient felt better without continuing omeprazole medication. Followup examinations of the patient were performed in the county hospital of Rottweil every year until the last examination in December 2008. The gastrin value was stable with 117 ng/L and a secretin provocation test was negative, demonstrating a biochemically cured disease up to now.

## 3. Discussion

Two pathological studies support the hypothesis that neuroendocrine cells exist in lymph nodes of the gastrinoma triangle in patients without a gastrinoma [7, 21].

The existence of primary lymph node gastrinoma as a cause of Zollinger-Ellison syndrome is, on the other hand, still controversial. In 1994, Arnold et al. analyzed 110 cases from 1982 to 1992 [22]. During surgical exploration, 21 patients had a disease limited to the lymph nodes. Exploration included intraoperative ultrasound, endoscopy, and explorative duodenotomy. All patients underwent a yearly biochemical and radiological followup. After 5.3 years, 9/21 (43%) patients remained biochemically cured. The other 12 patients showed a biochemically confirmed persistent or recurrent disease. In 2003, a prospective study of 176 patients with ZES was published. Only tumour-infiltrated lymph nodes were found in 45 cases. Postoperative gastrin determinations showed biochemical cure in 26/45 (57%) of the patients. During mean followup of 10.4 years 8/26 (31%) patients had a recurrent disease. Only 18/45 (40%) patients stayed disease-free. The recurrence rate in the 26 patients with tumour manifestation in more than lymph node did not differ from that in the 18 patients with one affected lymph node. According to this study, primary lymph node gastrinomas exist in 10% of the patients and are, therefore, not a rare entity [23]. Additionally, several case reports present patients with solitary lymph node tumour manifestation. In most cases, followup is too short to clarify the exclusive existence of primary lymph node gastrinoma or whether the lymph nodes are already metastases of very small undetected duodenal gastrinomas [21, 24, 25].

On the other hand, lymph node metastases have no influence on the overall survival of the patients with or without MEN-I. Also, 78% of the MEN-I patients and 52% of the patients with a sporadic gastrinoma show lymph node metastases at the time of surgical exploration [9]. In about 19–26%, only gastrinoma-positive lymph nodes are detected during explorative laparotomy, and despite extensive surgical treatment, no further tumour in the pancreas or the duodenum could be localized [5, 12, 26]. After the resection of the lymph nodes, postoperative gastrin levels normalized in 58–62% of these patients, and it is assumed that primary lymph node gastrinomas exist as presented in this case report.

Studies showed that the existence of liver metastasis decreases the overall 10-year survival rate from 96% to 26–30% [2, 27]. The existence of liver metastasis at first presentation differs between sporadic and MEN-I-associated gastrinomas (21–2%). The development of metachron liver metastases is equal in both entities [9]. Liver metastasis occurred more often with pancreatic than with duodenal tumours. In 21% of the patients, liver metastases already existed at the time of first diagnosis. The incidence of liver metastases is closely related to the size of primary tumours [9]. Primary tumours less than 1 cm show a prevalence of about 4%, tumours between 1 and 2 cm of 28% and tumours larger than 3 cm of 62% [27]. Patients, who undergo surgical resection of the primary tumour, have a significant risk reduction of developing liver metastasis, 3% versus 23%, compared to medically treated patients [27].

In light of the slow-growing tumour, a followup period less than 5 years seems to be too short to prove the existence of primary lymph node gastrinomas.

The aim of surgical exploration is to find and remove the primary tumour and all lymph node and distant metastases. If we suppose that primary lymph node gastrinoma does not exist, a curative therapy would be a partial pancreaticoduodenectomy including a radical lymphadenectomy. But is such an aggressive therapy justified, or should the patients be treated with a restricted procedure and a close-meshed followup? As is always the case with malignant diseases, patients can develop distant metastases, for example, in the liver, limiting the survival rate. On the other hand, a partial pancreaticoduodenectomy has a high morbidity rate. These days, a partial pancreaticoduodenectomy is a highly standardized operative procedure, but even in high-volume centres, the morbidity rate is still about 40% and the postoperative mortality rates vary between 1 and 6%. In over 2/3 of these cases, the complications are directly related to surgery. Pancreatic leakage with 2–14% and postoperative haemorrhage with 5% are the most common and severe complications [26, 28–32]. Long-term complications like diabetes mellitus exist in 27% of the patients undergoing a pancreatoduodenectomy [33]. Morbidity and mortality of extensive surgical exploration including Kocher's manoeuvre, duodenotomy, and resection of all known tumour lesions was reported with 11% and 0% in 1992 [13].

In our opinion, a prophylactic radical resection like a partial pancreatoduodenectomy with complete clearance of the gastrinoma triangle should not be performed due to the high morbidity and mortality. The tumour biology of gastrinomas allows a conservative procedure with resection of all known tumor locations and a medical followup in half-year intervals. In case of a postoperative persistent high gastrin level, a partial pancreaticoduodenectomy is probably the treatment of choice.

## 4. Conclusion

In several patients with a ZES, an explorative laparotomy does not detect the primary tumour in the duodenum or the pancreas but just a gastrinoma-positive lymph node. The existence of a primary lymph node gastrinoma is still controversial. The assumption of a very small primary tumour in the duodenal wall, which cannot be detected during surgery, suggests a partial pancreaticoduodenectomy as a curative treatment approach. The tumour biology allows a close-meshed followup and high postoperative morbidity and mortality of a partial pancreatoduodenectomy need not be accepted. After limited surgery, a long-term disease-free period is possible.
